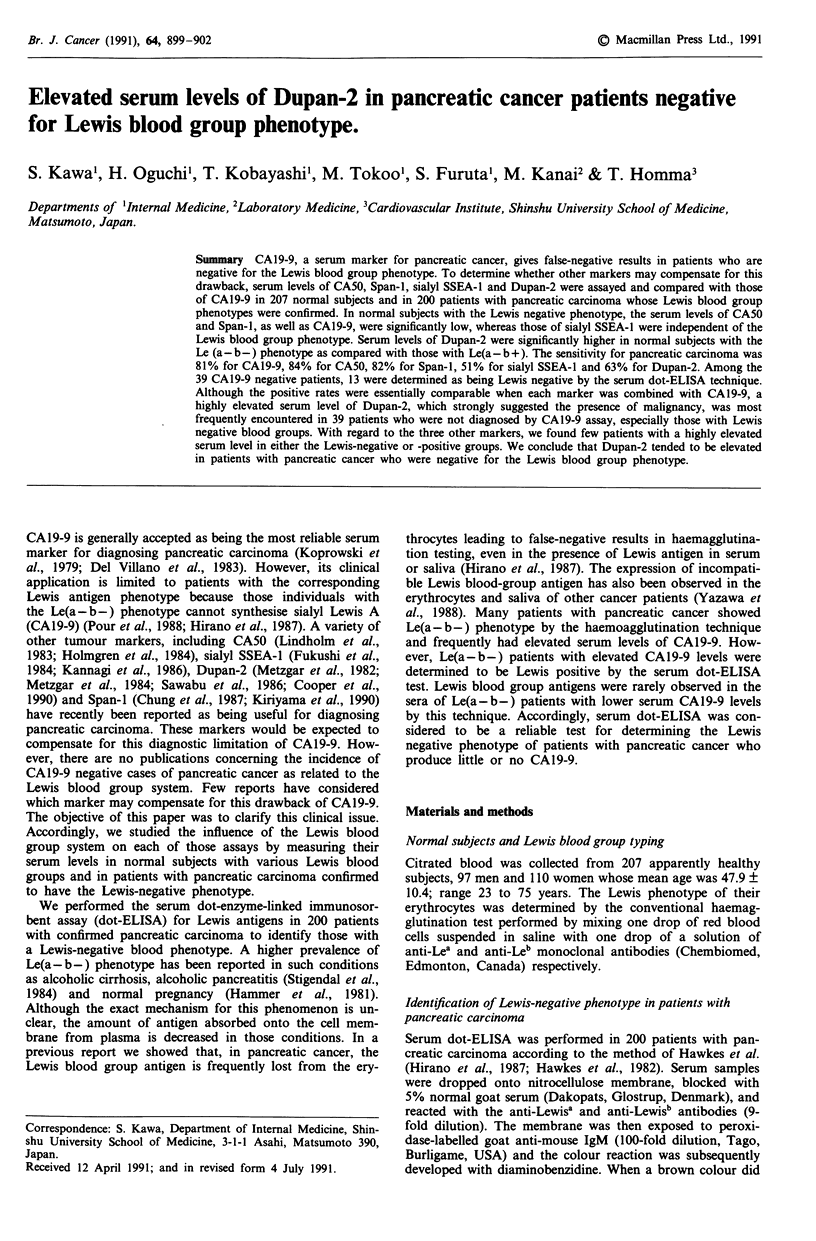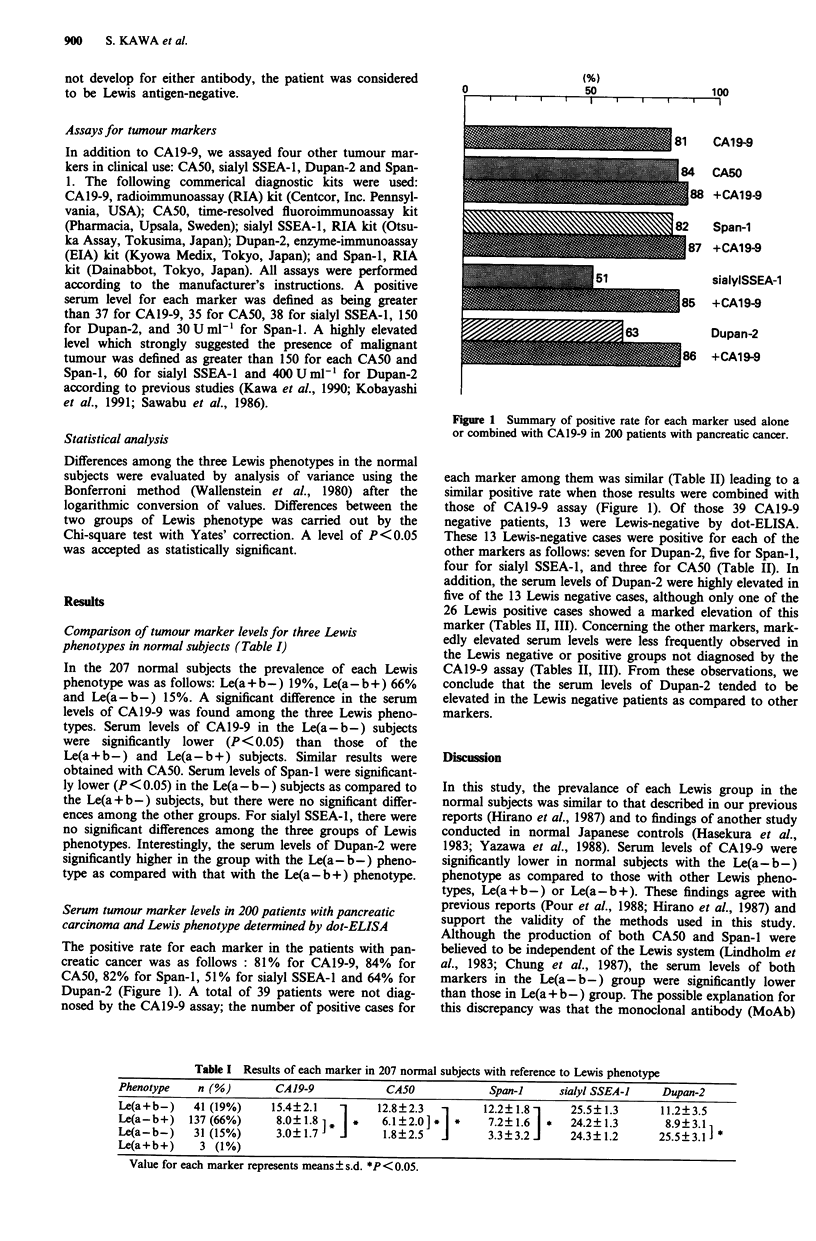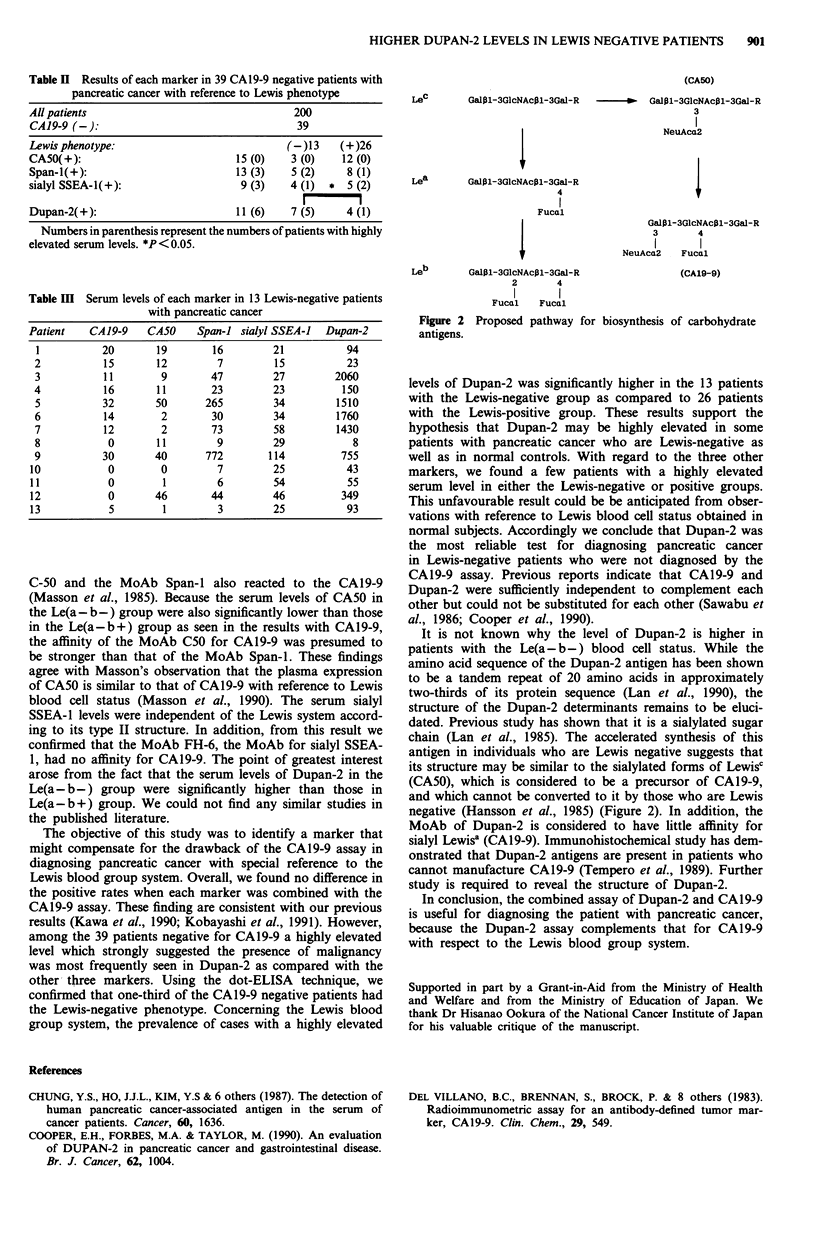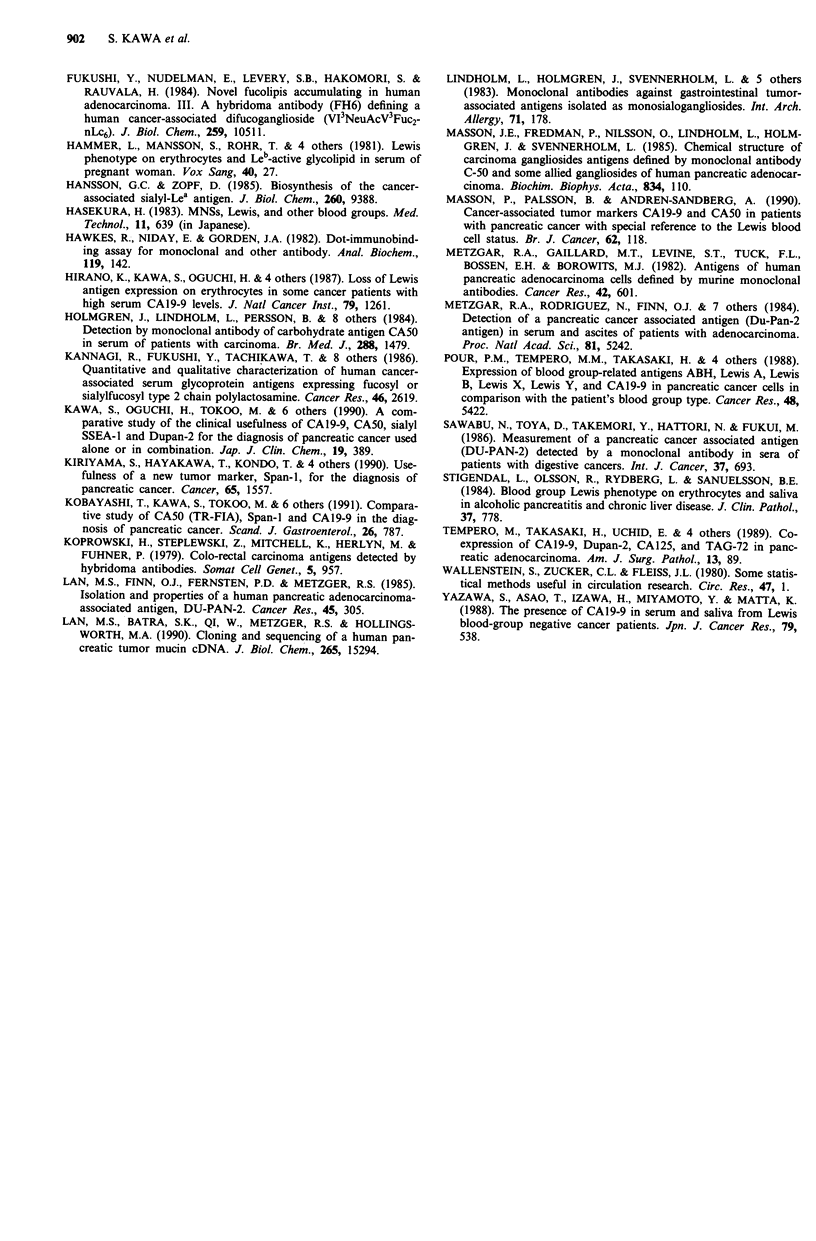# Elevated serum levels of Dupan-2 in pancreatic cancer patients negative for Lewis blood group phenotype.

**DOI:** 10.1038/bjc.1991.422

**Published:** 1991-11

**Authors:** S. Kawa, H. Oguchi, T. Kobayashi, M. Tokoo, S. Furuta, M. Kanai, T. Homma

**Affiliations:** Department of Internal Medicine, Shinshu University School of Medicine, Matsumoto, Japan.

## Abstract

CA19-9, a serum marker for pancreatic cancer, gives false-negative results in patients who are negative for the Lewis blood group phenotype. To determine whether other markers may compensate for this drawback, serum levels of CA50, Span-1, sialyl SSEA-1 and Dupan-2 were assayed and compared with those of CA19-9 in 207 normal subjects and in 200 patients with pancreatic carcinoma whose Lewis blood group phenotypes were confirmed. In normal subjects with the Lewis negative phenotype, the serum levels of CA50 and Span-1, as well as CA19-9, were significantly low, whereas those of sialyl SSEA-1 were independent of the Lewis blood group phenotype. Serum levels of Dupan-2 were significantly higher in normal subjects with the Le (a-b-) phenotype as compared with those with Le(a-b+). The sensitivity for pancreatic carcinoma was 81% for CA19-9, 84% for CA50, 82% for Span-1, 51% for sialyl SSEA-1 and 63% for Dupan-2. Among the 39 CA19-9 negative patients, 13 were determined as being Lewis negative by the serum dot-ELISA technique. Although the positive rates were essentially comparable when each marker was combined with CA19-9, a highly elevated serum level of Dupan-2, which strongly suggested the presence of malignancy, was most frequently encountered in 39 patients who were not diagnosed by CA19-9 assay, especially those with Lewis negative blood groups. With regard to the three other markers, we found few patients with a highly elevated serum level in either the Lewis-negative or -positive groups. We conclude that Dupan-2 tended to be elevated in patients with pancreatic cancer who were negative for the Lewis blood group phenotype.


Br. J. Cancer (1991), 64, 899-902 © Macmillan Press Ltd., 1991

### Elevated serum levels of Dupan-2 in pancreatic cancer patients negative for Lewis blood group phenotype.

S. Kawa[1], H. Oguchi[1], T. Kobayashi[1], M. Tokoo[1], S. Furuta[1], M. Kanai[2] & T. Homma[3]

*Departments of [1]Internal Medicine, [2]Laboratory Medicine, [3]Cardiovascular Institute, Shinshu University School of Medicine, Matsumoto, Japan.*

**Summary** CA19-9, a serum marker for pancreatic cancer, gives false-negative results in patients who are negative for the Lewis blood group phenotype. To determine whether other markers may compensate for this drawback, serum levels of CA50, Span-1, sialyl SSEA-1 and Dupan-2 were assayed and compared with those of CA19-9 in 207 normal subjects and in 200 patients with pancreatic carcinoma whose Lewis blood group phenotypes were confirmed. In normal subjects with the Lewis negative phenotype, the serum levels of CA50 and Span-1, as well as CA19-9, were significantly low, whereas those of sialyl SSEA-1 were independent of the Lewis blood group phenotype. Serum levels of Dupan-2 were significantly higher in normal subjects with the Le (a−b−) phenotype as compared with those with Le(a−b+). The sensitivity for pancreatic carcinoma was 81% for CA19-9, 84% for CA50, 82% for Span-1, 51% for sialyl SSEA-1 and 63% for Dupan-2. Among the 39 CA19-9 negative patients, 13 were determined as being Lewis negative by the serum dot-ELISA technique. Although the positive rates were essentially comparable when each marker was combined with CA19-9, a highly elevated serum level of Dupan-2, which strongly suggested the presence of malignancy, was most frequently encountered in 39 patients who were not diagnosed by CA19-9 assay, especially those with Lewis negative blood groups. With regard to the three other markers, we found few patients with a highly elevated serum level in either the Lewis-negative or -positive groups. We conclude that Dupan-2 tended to be elevated in patients with pancreatic cancer who were negative for the Lewis blood group phenotype.

CA19-9 is generally accepted as being the most reliable serum marker for diagnosing pancreatic carcinoma (Koprowski *et al.*, 1979; Del Villano *et al.*, 1983). However, its clinical application is limited to patients with the corresponding Lewis antigen phenotype because those individuals with the Le(a−b−) phenotype cannot synthesise sialyl Lewis A (CA19-9) (Pour *et al.*, 1988; Hirano *et al.*, 1987). A variety of other tumour markers, including CA50 (Lindholm *et al.*, 1983; Holmgren *et al.*, 1984), sialyl SSEA-1 (Fukushi *et al.*, 1984; Kannagi *et al.*, 1986), Dupan-2 (Metzgar *et al.*, 1982; Metzgar *et al.*, 1984; Sawabu *et al.*, 1986; Cooper *et al.*, 1990) and Span-1 (Chung *et al.*, 1987; Kiriyama *et al.*, 1990) have recently been reported as being useful for diagnosing pancreatic carcinoma. These markers would be expected to compensate for this diagnostic limitation of CA19-9. However, there are no publications concerning the incidence of CA19-9 negative cases of pancreatic cancer as related to the Lewis blood group system. Few reports have considered which marker may compensate for this drawback of CA19-9. The objective of this paper was to clarify this clinical issue. Accordingly, we studied the influence of the Lewis blood group system on each of those assays by measuring their serum levels in normal subjects with various Lewis blood groups and in patients with pancreatic carcinoma confirmed to have the Lewis-negative phenotype.

We performed the serum dot-enzyme-linked immunosorbent assay (dot-ELISA) for Lewis antigens in 200 patients with confirmed pancreatic carcinoma to identify those with a Lewis-negative blood phenotype. A higher prevalence of Le(a−b−) phenotype has been reported in such conditions as alcoholic cirrhosis, alcoholic pancreatitis (Stigendal *et al.*, 1984) and normal pregnancy (Hammer *et al.*, 1981). Although the exact mechanism for this phenomenon is unclear, the amount of antigen absorbed onto the cell membrane from plasma is decreased in those conditions. In a previous report we showed that, in pancreatic cancer, the Lewis blood group antigen is frequently lost from the erythrocytes leading to false-negative results in haemagglutination testing, even in the presence of Lewis antigen in serum or saliva (Hirano *et al.*, 1987). The expression of incompatible Lewis blood-group antigen has also been observed in the erythrocytes and saliva of other cancer patients (Yazawa *et al.*, 1988). Many patients with pancreatic cancer showed Le(a−b−) phenotype by the haemoagglutination technique and frequently had elevated serum levels of CA19-9. However, Le(a−b−) patients with elevated CA19-9 levels were determined to be Lewis positive by the serum dot-ELISA test. Lewis blood group antigens were rarely observed in the sera of Le(a−b−) patients with lower serum CA19-9 levels by this technique. Accordingly, serum dot-ELISA was considered to be a reliable test for determining the Lewis negative phenotype of patients with pancreatic cancer who produce little or no CA19-9.

Correspondence: S. Kawa, Department of Internal Medicine, Shinshu University School of Medicine, 3-1-1 Asahi, Matsumoto 390, Japan.
Received 12 April 1991; and in revised form 4 July 1991.

### Materials and methods

### *Normal subjects and Lewis blood group typing*

Citrated blood was collected from 207 apparently healthy subjects, 97 men and 110 women whose mean age was 47.9 ± 10.4; range 23 to 75 years. The Lewis phenotype of their erythrocytes was determined by the conventional haemagglutination test performed by mixing one drop of red blood cells suspended in saline with one drop of a solution of anti-$Le^a$ and anti-$Le^b$ monoclonal antibodies (Chembiomed, Edmonton, Canada) respectively.

### *Identification of Lewis-negative phenotype in patients with pancreatic carcinoma*

Serum dot-ELISA was performed in 200 patients with pancreatic carcinoma according to the method of Hawkes *et al.* (Hirano *et al.*, 1987; Hawkes *et al.*, 1982). Serum samples were dropped onto nitrocellulose membrane, blocked with 5% normal goat serum (Dakopats, Glostrup, Denmark), and reacted with the anti-$Lewis^a$ and anti-$Lewis^b$ antibodies (9-fold dilution). The membrane was then exposed to peroxidase-labelled goat anti-mouse IgM (100-fold dilution, Tago, Burligame, USA) and the colour reaction was subsequently developed with diaminobenzidine. When a brown colour did not develop for either antibody, the patient was considered to be Lewis antigen-negative.

### *Assays for tumour markers*

In addition to CA19-9, we assayed four other tumour markers in clinical use: CA50, sialyl SSEA-1, Dupan-2 and Span-1. The following commerical diagnostic kits were used: CA19-9, radioimmunoassay (RIA) kit (Centcor, Inc. Pennsylvania, USA); CA50, time-resolved fluoroimmunoassay kit (Pharmacia, Upsala, Sweden); sialyl SSEA-1, RIA kit (Otsuka Assay, Tokusima, Japan); Dupan-2, enzyme-immunoassay (EIA) kit (Kyowa Medix, Tokyo, Japan); and Span-1, RIA kit (Dainabbot, Tokyo, Japan). All assays were performed according to the manufacturer's instructions. A positive serum level for each marker was defined as being greater than 37 for CA19-9, 35 for CA50, 38 for sialyl SSEA-1, 150 for Dupan-2, and 30 U $ml^{-1}$ for Span-1. A highly elevated level which strongly suggested the presence of malignant tumour was defined as greater than 150 for each CA50 and Span-1, 60 for sialyl SSEA-1 and 400 U $ml^{-1}$ for Dupan-2 according to previous studies (Kawa *et al.*, 1990; Kobayashi *et al.*, 1991; Sawabu *et al.*, 1986).

### *Statistical analysis*

Differences among the three Lewis phenotypes in the normal subjects were evaluated by analysis of variance using the Bonferroni method (Wallenstein *et al.*, 1980) after the logarithmic conversion of values. Differences between the two groups of Lewis phenotype was carried out by the Chi-square test with Yates' correction. A level of $P<0.05$ was accepted as statistically significant.

### Results

### *Comparison of tumour marker levels for three Lewis phenotypes in normal subjects (Table I)*

In the 207 normal subjects the prevalence of each Lewis phenotype was as follows: Le(a+b−) 19%, Le(a−b+) 66% and Le(a−b−) 15%. A significant difference in the serum levels of CA19-9 was found among the three Lewis phenotypes. Serum levels of CA19-9 in the Le(a−b−) subjects were significantly lower ($P<0.05$) than those of the Le(a+b−) and Le(a−b+) subjects. Similar results were obtained with CA50. Serum levels of Span-1 were significantly lower ($P<0.05$) in the Le(a−b−) subjects as compared to the Le(a+b−) subjects, but there were no significant differences among the other groups. For sialyl SSEA-1, there were no significant differences among the three groups of Lewis phenotypes. Interestingly, the serum levels of Dupan-2 were significantly higher in the group with the Le(a−b−) phenotype as compared with that with the Le(a−b+) phenotype.

### *Serum tumour marker levels in 200 patients with pancreatic carcinoma and Lewis phenotype determined by dot-ELISA*

The positive rate for each marker in the patients with pancreatic cancer was as follows : 81% for CA19-9, 84% for CA50, 82% for Span-1, 51% for sialyl SSEA-1 and 64% for Dupan-2 (Figure 1). A total of 39 patients were not diagnosed by the CA19-9 assay; the number of positive cases for each marker among them was similar (Table II) leading to a similar positive rate when those results were combined with those of CA19-9 assay (Figure 1). Of those 39 CA19-9 negative patients, 13 were Lewis-negative by dot-ELISA. These 13 Lewis-negative cases were positive for each of the other markers as follows: seven for Dupan-2, five for Span-1, four for sialyl SSEA-1, and three for CA50 (Table II). In addition, the serum levels of Dupan-2 were highly elevated in five of the 13 Lewis negative cases, although only one of the 26 Lewis positive cases showed a marked elevation of this marker (Tables II, III). Concerning the other markers, markedly elevated serum levels were less frequently observed in the Lewis negative or positive groups not diagnosed by the CA19-9 assay (Tables II, III). From these observations, we conclude that the serum levels of Dupan-2 tended to be elevated in the Lewis negative patients as compared to other markers.

**Figure 1** Summary of positive rate for each marker used alone or combined with CA19-9 in 200 patients with pancreatic cancer.

### Discussion

In this study, the prevalance of each Lewis group in the normal subjects was similar to that described in our previous reports (Hirano *et al.*, 1987) and to findings of another study conducted in normal Japanese controls (Hasekura *et al.*, 1983; Yazawa *et al.*, 1988). Serum levels of CA19-9 were significantly lower in normal subjects with the Le(a−b−) phenotype as compared to those with other Lewis phenotypes, Le(a+b−) or Le(a−b+). These findings agree with previous reports (Pour *et al.*, 1988; Hirano *et al.*, 1987) and support the validity of the methods used in this study. Although the production of both CA50 and Span-1 were believed to be independent of the Lewis system (Lindholm *et al.*, 1983; Chung *et al.*, 1987), the serum levels of both markers in the Le(a−b−) group were significantly lower than those in Le(a+b−) group. The possible explanation for this discrepancy was that the monoclonal antibody (MoAb)

**Table I** Results of each marker in 207 normal subjects with reference to Lewis phenotype

| *Phenotype* | *n (%)* | *CA19-9* | *CA50* | *Span-1* | *sialyl SSEA-1* | *Dupan-2* |
|---|---|---|---|---|---|---|
| Le(a+b−) | 41 (19%) | 15.4±2.1 | 12.8±2.3 | 12.2±1.8 | 25.5±1.3 | 11.2±3.5 |
| Le(a−b+) | 137 (66%) | 8.0±1.8 ]* ]* | 6.1±2.0 ]* ]* | 7.2±1.6 ]* | 24.2±1.3 | 8.9±3.1 |
| Le(a−b−) | 31 (15%) | 3.0±1.7 | 1.8±2.5 | 3.3±3.2 | 24.3±1.2 | 25.5±3.1 ]* |
| Le(a+b+) | 3 (1%) | | | | | |

Value for each marker represents means±s.d. $^*P<0.05$.

**Table II** Results of each marker in 39 CA19-9 negative patients with pancreatic cancer with reference to Lewis phenotype

| | | | |
|---|---|---|---|
| *All patients* | | 200 | |
| *CA19-9 (−):* | | 39 | |
| *Lewis phenotype:* | | (−)13 | (+)26 |
| CA50(+): | 15 (0) | 3 (0) | 12 (0) |
| Span-1(+): | 13 (3) | 5 (2) | 8 (1) |
| sialyl SSEA-1(+): | 9 (3) | 4 (1) | * 5 (2) |
| Dupan-2(+): | 11 (6) | 7 (5) | 4 (1) |

Numbers in parenthesis represent the numbers of patients with highly elevated serum levels. *$P<0.05$.

**Table III** Serum levels of each marker in 13 Lewis-negative patients with pancreatic cancer

| *Patient* | *CA19-9* | *CA50* | *Span-1* | *sialyl SSEA-1* | *Dupan-2* |
|---|---|---|---|---|---|
| 1 | 20 | 19 | 16 | 21 | 94 |
| 2 | 15 | 12 | 7 | 15 | 23 |
| 3 | 11 | 9 | 47 | 27 | 2060 |
| 4 | 16 | 11 | 23 | 23 | 150 |
| 5 | 32 | 50 | 265 | 34 | 1510 |
| 6 | 14 | 2 | 30 | 34 | 1760 |
| 7 | 12 | 2 | 73 | 58 | 1430 |
| 8 | 0 | 11 | 9 | 29 | 8 |
| 9 | 30 | 40 | 772 | 114 | 755 |
| 10 | 0 | 0 | 7 | 25 | 43 |
| 11 | 0 | 1 | 6 | 54 | 55 |
| 12 | 0 | 46 | 44 | 46 | 349 |
| 13 | 5 | 1 | 3 | 25 | 93 |

C-50 and the MoAb Span-1 also reacted to the CA19-9 (Masson *et al.*, 1985). Because the serum levels of CA50 in the Le(a−b−) group were also significantly lower than those in the Le(a−b+) group as seen in the results with CA19-9, the affinity of the MoAb C50 for CA19-9 was presumed to be stronger than that of the MoAb Span-1. These findings agree with Masson's observation that the plasma expression of CA50 is similar to that of CA19-9 with reference to Lewis blood cell status (Masson *et al.*, 1990). The serum sialyl SSEA-1 levels were independent of the Lewis system according to its type II structure. In addition, from this result we confirmed that the MoAb FH-6, the MoAb for sialyl SSEA-1, had no affinity for CA19-9. The point of greatest interest arose from the fact that the serum levels of Dupan-2 in the Le(a−b−) group were significantly higher than those in Le(a−b+) group. We could not find any similar studies in the published literature.

The objective of this study was to identify a marker that might compensate for the drawback of the CA19-9 assay in diagnosing pancreatic cancer with special reference to the Lewis blood group system. Overall, we found no difference in the positive rates when each marker was combined with the CA19-9 assay. These finding are consistent with our previous results (Kawa *et al.*, 1990; Kobayashi *et al.*, 1991). However, among the 39 patients negative for CA19-9 a highly elevated level which strongly suggested the presence of malignancy was most frequently seen in Dupan-2 as compared with the other three markers. Using the dot-ELISA technique, we confirmed that one-third of the CA19-9 negative patients had the Lewis-negative phenotype. Concerning the Lewis blood group system, the prevalence of cases with a highly elevated

**Figure 2** Proposed pathway for biosynthesis of carbohydrate antigens.

levels of Dupan-2 was significantly higher in the 13 patients with the Lewis-negative group as compared to 26 patients with the Lewis-positive group. These results support the hypothesis that Dupan-2 may be highly elevated in some patients with pancreatic cancer who are Lewis-negative as well as in normal controls. With regard to the three other markers, we found a few patients with a highly elevated serum level in either the Lewis-negative or positive groups. This unfavourable result could be be anticipated from observations with reference to Lewis blood cell status obtained in normal subjects. Accordingly we conclude that Dupan-2 was the most reliable test for diagnosing pancreatic cancer in Lewis-negative patients who were not diagnosed by the CA19-9 assay. Previous reports indicate that CA19-9 and Dupan-2 were sufficiently independent to complement each other but could not be substituted for each other (Sawabu *et al.*, 1986; Cooper *et al.*, 1990).

It is not known why the level of Dupan-2 is higher in patients with the Le(a−b−) blood cell status. While the amino acid sequence of the Dupan-2 antigen has been shown to be a tandem repeat of 20 amino acids in approximately two-thirds of its protein sequence (Lan *et al.*, 1990), the structure of the Dupan-2 determinants remains to be elucidated. Previous study has shown that it is a sialylated sugar chain (Lan *et al.*, 1985). The accelerated synthesis of this antigen in individuals who are Lewis negative suggests that its structure may be similar to the sialylated forms of Lewis$^c$ (CA50), which is considered to be a precursor of CA19-9, and which cannot be converted to it by those who are Lewis negative (Hansson *et al.*, 1985) (Figure 2). In addition, the MoAb of Dupan-2 is considered to have little affinity for sialyl Lewis$^a$ (CA19-9). Immunohistochemical study has demonstrated that Dupan-2 antigens are present in patients who cannot manufacture CA19-9 (Tempero *et al.*, 1989). Further study is required to reveal the structure of Dupan-2.

In conclusion, the combined assay of Dupan-2 and CA19-9 is useful for diagnosing the patient with pancreatic cancer, because the Dupan-2 assay complements that for CA19-9 with respect to the Lewis blood group system.

Supported in part by a Grant-in-Aid from the Ministry of Health and Welfare and from the Ministry of Education of Japan. We thank Dr Hisanao Ookura of the National Cancer Institute of Japan for his valuable critique of the manuscript.

### References

CHUNG, Y.S., HO, J.J.L., KIM, Y.S & 6 others (1987). The detection of human pancreatic cancer-associated antigen in the serum of cancer patients. *Cancer*, **60**, 1636.

COOPER, E.H., FORBES, M.A. & TAYLOR, M. (1990). An evaluation of DUPAN-2 in pancreatic cancer and gastrointestinal disease. *Br. J. Cancer*, **62**, 1004.

DEL VILLANO, B.C., BRENNAN, S., BROCK, P. & 8 others (1983). Radioimmunometric assay for an antibody-defined tumor marker, CA19-9. *Clin. Chem.*, **29**, 549.

FUKUSHI, Y., NUDELMAN, E., LEVERY, S.B., HAKOMORI, S. & RAUVALA, H. (1984). Novel fucolipis accumulating in human adenocarcinoma. III. A hybridoma antibody (FH6) defining a human cancer-associated difucoganglioside ($VI^3NeuAcV^3Fuc_2$-$nLc_6$). *J. Biol. Chem.*, **259**, 10511.

HAMMER, L., MANSSON, S., ROHR, T. & 4 others (1981). Lewis phenotype on erythrocytes and $Le^b$-active glycolipid in serum of pregnant woman. *Vox Sang*, **40**, 27.

HANSSON, G.C. & ZOPF, D. (1985). Biosynthesis of the cancer-associated sialyl-$Le^a$ antigen. *J. Biol. Chem.*, **260**, 9388.

HASEKURA, H. (1983). MNSs, Lewis, and other blood groups. *Med. Technol.*, **11**, 639 (in Japanese).

HAWKES, R., NIDAY, E. & GORDEN, J.A. (1982). Dot-immunobinding assay for monoclonal and other antibody. *Anal. Biochem.*, **119**, 142.

HIRANO, K., KAWA, S., OGUCHI, H. & 4 others (1987). Loss of Lewis antigen expression on erythrocytes in some cancer patients with high serum CA19-9 levels. *J. Natl Cancer Inst.*, **79**, 1261.

HOLMGREN, J., LINDHOLM, L., PERSSON, B. & 8 others (1984). Detection by monoclonal antibody of carbohydrate antigen CA50 in serum of patients with carcinoma. *Br. Med. J.*, **288**, 1479.

KANNAGI, R., FUKUSHI, Y., TACHIKAWA, T. & 8 others (1986). Quantitative and qualitative characterization of human cancer-associated serum glycoprotein antigens expressing fucosyl or sialylfucosyl type 2 chain polylactosamine. *Cancer Res.*, **46**, 2619.

KAWA, S., OGUCHI, H., TOKOO, M. & 6 others (1990). A comparative study of the clinical usefulness of CA19-9, CA50, sialyl SSEA-1 and Dupan-2 for the diagnosis of pancreatic cancer used alone or in combination. *Jap. J. Clin. Chem.*, **19**, 389.

KIRIYAMA, S., HAYAKAWA, T., KONDO, T. & 4 others (1990). Usefulness of a new tumor marker, Span-1, for the diagnosis of pancreatic cancer. *Cancer*, **65**, 1557.

KOBAYASHI, T., KAWA, S., TOKOO, M. & 6 others (1991). Comparative study of CA50 (TR-FIA), Span-1 and CA19-9 in the diagnosis of pancreatic cancer. *Scand. J. Gastroenterol.*, **26**, 787.

KOPROWSKI, H., STEPLEWSKI, Z., MITCHELL, K., HERLYN, M. & FUHNER, P. (1979). Colo-rectal carcinoma antigens detected by hybridoma antibodies. *Somat Cell Genet.*, **5**, 957.

LAN, M.S., FINN, O.J., FERNSTEN, P.D. & METZGER, R.S. (1985). Isolation and properties of a human pancreatic adenocarcinoma-associated antigen, DU-PAN-2. *Cancer Res.*, **45**, 305.

LAN, M.S., BATRA, S.K., QI, W., METZGER, R.S. & HOLLINGSWORTH, M.A. (1990). Cloning and sequencing of a human pancreatic tumor mucin cDNA. *J. Biol. Chem.*, **265**, 15294.

LINDHOLM, L., HOLMGREN, J., SVENNERHOLM, L. & 5 others (1983). Monoclonal antibodies against gastrointestinal tumor-associated antigens isolated as monosialogangliosides. *Int. Arch. Allergy*, **71**, 178.

MASSON, J.E., FREDMAN, P., NILSSON, O., LINDHOLM, L., HOLMGREN, J. & SVENNERHOLM, L. (1985). Chemical structure of carcinoma gangliosides antigens defined by monoclonal antibody C-50 and some allied gangliosides of human pancreatic adenocarcinoma. *Biochim. Biophys. Acta.*, **834**, 110.

MASSON, P., PALSSON, B. & ANDREN-SANDBERG, A. (1990). Cancer-associated tumor markers CA19-9 and CA50 in patients with pancreatic cancer with special reference to the Lewis blood cell status. *Br. J. Cancer*, **62**, 118.

METZGAR, R.A., GAILLARD, M.T., LEVINE, S.T., TUCK, F.L., BOSSEN, E.H. & BOROWITS, M.J. (1982). Antigens of human pancreatic adenocarcinoma cells defined by murine monoclonal antibodies. *Cancer Res.*, **42**, 601.

METZGAR, R.A., RODRIGUEZ, N., FINN, O.J. & 7 others (1984). Detection of a pancreatic cancer associated antigen (Du-Pan-2 antigen) in serum and ascites of patients with adenocarcinoma. *Proc. Natl Acad. Sci.*, **81**, 5242.

POUR, P.M., TEMPERO, M.M., TAKASAKI, H. & 4 others (1988). Expression of blood group-related antigens ABH, Lewis A, Lewis B, Lewis X, Lewis Y, and CA19-9 in pancreatic cancer cells in comparison with the patient's blood group type. *Cancer Res.*, **48**, 5422.

SAWABU, N., TOYA, D., TAKEMORI, Y., HATTORI, N. & FUKUI, M. (1986). Measurement of a pancreatic cancer associated antigen (DU-PAN-2) detected by a monoclonal antibody in sera of patients with digestive cancers. *Int. J. Cancer*, **37**, 693.

STIGENDAL, L., OLSSON, R., RYDBERG, L. & SANUELSSON, B.E. (1984). Blood group Lewis phenotype on erythrocytes and saliva in alcoholic pancreatitis and chronic liver disease. *J. Clin. Pathol.*, **37**, 778.

TEMPERO, M., TAKASAKI, H., UCHID, E. & 4 others (1989). Co-expression of CA19-9, Dupan-2, CA125, and TAG-72 in pancreatic adenocarcinoma. *Am. J. Surg. Pathol.*, **13**, 89.

WALLENSTEIN, S., ZUCKER, C.L. & FLEISS, J.L. (1980). Some statistical methods useful in circulation research. *Circ. Res.*, **47**, 1.

YAZAWA, S., ASAO, T., IZAWA, H., MIYAMOTO, Y. & MATTA, K. (1988). The presence of CA19-9 in serum and saliva from Lewis blood-group negative cancer patients. *Jpn. J. Cancer Res.*, **79**, 538.